# First record of multi-species synchronous coral spawning from Malaysia

**DOI:** 10.7717/peerj.777

**Published:** 2015-02-17

**Authors:** Alvin Chelliah, Halimi Bin Amar, Julian Hyde, Katie Yewdall, Peter D. Steinberg, James R. Guest

**Affiliations:** 1 Reef Check Malaysia, Jalan Ampang, Kuala Lumpur, Malaysia; 2 Tioman Dive Centre, Kampong Tekek, Tioman Island, Pahang, Malaysia; 3 Centre for Marine Bio-Innovation, School of Biological, Earth and Environmental Sciences, University of New South Wales, Sydney, NSW, Australia; 4 Advanced Environmental Biotechnology Centre, Nanyang Environment and Water Research Institute, Nanyang Technological University, Singapore, Singapore; 5 Sydney Institute of Marine Science, Mosman, NSW, Australia

**Keywords:** Multi-species spawning, Coral reef, Pulau Tioman

## Abstract

Knowledge about the timing and synchrony of coral spawning has important implications for both the ecology and management of coral reef ecosystems. Data on the timing of spawning and extent of synchrony, however, are still lacking for many coral reefs, particularly from equatorial regions and from locations within the coral triangle. Here we present the first documentation of a multi-species coral spawning event from reefs around Pulau Tioman, Peninsular Malaysia, a popular diving and tourist destination located on the edge of the coral triangle. At least 8 coral species from 3 genera (*Acropora*, *Montipora* and *Porites*) participated in multi-species spawning over five nights in April 2014, between two nights before and two nights after the full moon. In addition, two *Acropora* species were witnessed spawning one night prior to the full moon in October 2014. While two of the *Acropora* species that reproduced in April (*A. millepora* and *A. nasuta*) exhibited highly synchronous spawning (100% of sampled colonies), two other common species (*A. hyacinthus* and *A. digitifera*) did not contain visible eggs in the majority of colonies sampled (i.e., <15% of colonies) in either April or October, suggesting that these species spawn at other times of the year. To the best of our knowledge, this is the first detailed documented observation of multi-species coral spawning from reefs in Malaysia. These data provide further support for the contention that this phenomenon is a feature of all speciose coral assemblages, including equatorial reefs. More research is needed, however, to determine the seasonal cycles and extent of spawning synchrony on these reefs and elsewhere in Malaysia.

## Introduction

Knowledge about the timing and synchrony of coral spawning has important implications for both the ecology and management of coral reef ecosystems (Guest, 2008). The majority of scleractinian corals (i.e., >60% of species) are hermaphrodites that broadcast gametes for external fertilization (Baird, Guest & Willis, 2009). Broadcast spawning usually occurs annually and can be highly synchronised within populations (Harrison & Wallace, 1990). In addition, within diverse coral assemblages there is often considerable overlap in spawning times among species, leading to extensive multi-species spawning events involving numerous taxa (Babcock et al., 1986). For years it was thought that these remarkable reproductive events were restricted to geographical regions that experience large annual fluctuations in temperature and irradiance (Oliver et al., 1988). More recent research from a wide range of locations, however, has revealed that multi-species coral spawning is likely to be a feature of all speciose coral assemblages (Guest et al., 2005a; Baird, Guest & Willis, 2009; Bouwmeester et al., 2014). A plausible explanation for ubiquitous multi-species spawning can be summarized as follows: Spawning synchrony within broadcast spawning species, driven by external environmental timing cues, is likely to be highly adaptive as it increases the chances of cross fertilization (Babcock et al., 1986; Oliver & Babcock, 1992). Sympatric coral species are likely to respond independently but in a similar manner to the available timing cues—which will lead to overlap in spawning times among species in speciose assemblages (Oliver et al., 1988). Considering that seasonal timing cues (e.g., sea temperature and irradiance) are features of all coastal locations (even at the equator) (Guest et al., 2005a), multi-species spawning is also likely to be a feature of all speciose coral assemblages.

Despite recent advances in knowledge from several previously understudied locations (e.g., Vicentuan et al., 2008; Permata et al., 2012), data on spawning timing and extent of synchrony are still lacking for many coral reefs, particularly from locations within the coral triangle, an area of exceptionally high species diversity encompassing Malaysia, Indonesia, the Philippines and New Guinea (Hoeksema, 2007). The east coast of Peninsular Malaysia has almost 400 recorded scleractinian species, around 70% of species recorded from the entire coral triangle (Huang et al., 2014), and is therefore an area of considerable global importance in terms of marine biodiversity (Harborne et al., 2000). Here we present the first documentation of a multi-species coral spawning event from reefs around Pulau Tioman, Peninsular Malaysia (2°49′09.39″N, 104° 09′ 34.26″E), a popular diving and tourist destination located on the edge of the coral triangle.

## Materials and Methods

Evidence from reefs within the coral triangle suggest two coral spawning peaks in March/April and October/November, typically with a minor and a major spawning season for each location (Baird, Guest & Willis, 2009). The spawning times of coral species at sites around Pulau Tioman were examined using three different methods. Firstly, corals were sampled at two fringing reef sites on the west coast of Tioman (TDC House Reef: 2°48′56.47″N 104°09′05.66″E and; Tumuk: 2°47′32.80″N 104°07′22.02″E) on April 12 2014 (3 days before the full moon) and on October 7 2014 (1 day before full moon) to establish the extent of population synchrony within selected coral populations of *Acropora*. Sampling was done by removing up to three branches from randomly selected, independent (i.e., >5 m apart), replicate colonies of *Acropora millepora*, *A. nasuta*, *A. hyacinthus* and *A. digitifera* (Table 1) (following Baird, Marshall & Wolstenholme, 2002). Only *Acropora* colonies >30 cm diameter were sampled to ensure that all were of sufficient size and age to be reproductively mature (see Baria et al., 2012; Wallace, 1985). *A. millepora*, and *A. nasuta* were only sampled in April whereas *A. hyacinthus*, *A. digitifera* were sampled in April and October. For each colony, the presence or absence of visible pigmented or white eggs was noted *in situ* by a snorkeler. The presence of pigmented oocytes is indicative of spawning on or close to the date of the next full moon, whereas the presence of visible white eggs indicates that colony will spawn within the next two to three months. Empty colonies have either recently spawned or will not spawn for at least three months (Baird, Marshall & Wolstenholme, 2002). Secondly, to establish the night and time of spawning and the extent of spawning synchrony, we placed small egg-sperm bundle traps made from the bases of upturned plastic water bottles (Guest et al., 2010) over 12 gravid colonies of *A. millepora* and eight of *A. nasuta* on 12 April 2014 at TDC House Reef. Gamete traps were also placed over 2 colonies of *A. digitifera* and, in addition, 2 colonies of *A. tenuis* that were found to contain pigmented eggs on 7 October 2014. Traps were checked each morning for the presence or absence of released gametes until all colonies had spawned. Finally, *in situ* observations were made at TDC House Reef by snorkelers on the nights of 13 to 17 April 2014 and on 8 and 9 October 2014 between the hours of 1900 and 2300 to document spawning (approx. 28 h of *in situ* observation time in total). While the main aim of the direct observations was to establish the timing of spawning of the tagged *Acropora* colonies, a note was also made of any other coral species spawning during the observation period to assess the extent multi-species spawning at this site.

**Table 1:** Proportion of colonies sampled containing visible eggs. Proportion of population with pigmented eggs, white eggs and/or empty colonies in April and October 2014.

Species	Date	Pigmented (%)	White (%)	Empty (%)	*n*
*Acropora millepora*	12/04/2014	100	0	0	26
*Acropora nasuta*	12/04/2014	100	0	0	17
*Acropora digitifera*	12/04/2014	0	0	100	20
	7/10/2014	14	0	0	15
*Acropora hyacinthus*	12/04/2014	5	0	0	20
	7/10/2014	0	0	100	15

## Results and Discussion

**Figure 1:**
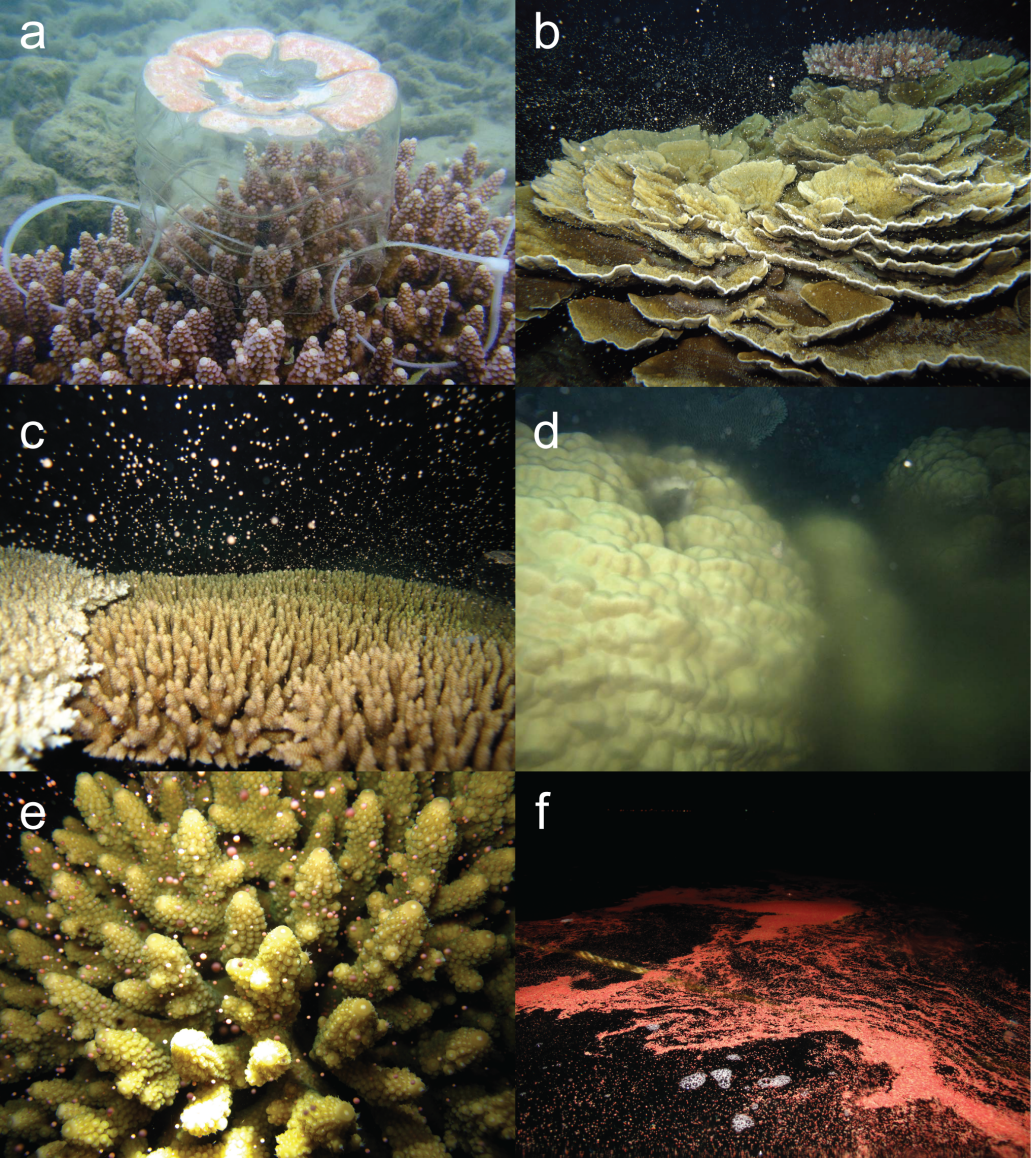
Multi species coral spawning in Pulau Tioman. Images of coral spawning in Pulau Tioman showing: (A) a gamete trap containing egg-sperm bundles on *Acropora digitifera*, (B) spawning of *Montipora* sp. 1, (C) *A. millepora*, (D) *Porites* sp. 2, (E) *A. humilis*, (F) and a gamete slick on the surface immediately after spawning. (Photos by Alvin Chelliah)

All sampled colonies of *A. millepora* and *A. nasuta* contained visible pigmented eggs when sampled on 12 April 2014 (Table 1). In contrast all sampled colonies of *A. digitifera* were empty of eggs in April and only 5% of *A. hyacinthus* colonies contained pigmented eggs in April, with the remainder of the sampled colonies being empty (Table 1). In October, all *A. hyacinthus* colonies were empty whereas 14% of *A. digitifera* colonies contained pigmented eggs (Table 1). Examination of the gamete traps showed that 2 colonies (17%) of *A. millepora* colonies spawned on 13 April, while the remaining tagged colonies of both *A. millepora* and *A. nasuta* spawned on 14 April (one night before the full moon) (Fig. 1 and Table 2). Similarly, on October 8, egg-sperm bundles were found in gamete traps placed over two tagged colonies each of *A. tenuis* and *A. digitifera*, indicating spawning on October 7 (one night before full moon) for these species. Coral spawning was observed *in situ* on four of the five nights of observation in April (13, 14, 16 and 17 April) between the hours of 2030 and 2225. No corals were observed spawning on April 15. At least 8 species from 3 genera (*Acropora*, *Montipora* and *Porites*) and 2 families participated in the spawning event (Fig. 1 and Table 2). Night time observations were carried out on October 8 and 9, but no spawning was witnessed directly on these nights. To the best of our knowledge, this is the first detailed documented observation of multi-species coral spawning from reefs in Malaysia. Our data, therefore, support the contention that this phenomenon is a feature of all speciose coral assemblages, including those on equatorial reefs (Baird & Guest, 2009; Guest et al., 2005a). The number of species observed to participate in these events is relatively modest compared to spawning events seen elsewhere (e.g., Babcock et al., 1986). Considering, however, that Pulau Tioman has at least 180 known coral species (Harborne et al., 2000) and that observations were only carried out at one site by two or three observers, it is likely that more extensive surveys will reveal other species participating in these multi-species spawning events.

**Table 2:** Species participation and timing of spawning during multi-species spawning in Pulau Tioman. Species participation during a multi-species spawning event in April 2014. Spawning nights are relative to date of full moon in 2014 (April 15).

Family	Species	Spawning nights	Spawning time	Gametes released
Acroporidae	*Acropora millepora*	−2 to −1	2115 to 2200	B
	*Acropora nasuta*	−1	2115 to 2200	B
	*Acropora humilis*	−1	2115 to 2200	B
	*Acropora valida*	−1	2115 to 2200	B
	*Montipora* sp. 1	+ 1 to +2	2030 to 2225	B
	*Montipora* sp. 2	+ 2	2030 to 2225	B
Poritidae	*Porites* sp. 1	+ 1 to +2	2030 to 2225	S
	*Porites* sp. 2	+ 1	2115 to 2225	S

**Notes.**

Type of gamete release B eggsperm bundles S sperm

While two species of *Acropora* (*A. millepora* and *A. nasuta*) exhibited highly synchronous spawning in April, two other common species (*A. hyacinthus* and *A. digitifera*) did not contain visible eggs in the majority of colonies sampled in either April or October, suggesting spawning at other times of the year for these species. While evidence from nearby locations suggest that March/April and October/November are the two main spawning peaks for this biogeographic region (Baird, Guest & Willis, 2009) extended spawning lasting several months are common on many Indo-Pacific coral reefs (e.g., Bouwmeester et al., 2014). The seasonal timing of spawning (i.e., March/April) for *A. millepora* and *A. nasuta* is consistent with observations of spawning seasonality for *Acropora* species from other locations in Southeast Asia (e.g., Singapore, north-western Philippines, Indonesia) (Guest et al., 2002; Vicentuan et al., 2008; Permata et al., 2012). Seasonal spawning timing within and among coral species is often consistent over broad geographical ranges because individuals are likely to respond in a similar way to environmental timing cues (e.g., sea surface temperature, irradiance) (Willis et al., 1985; Van Woesik, Lacharmoise & Köksal, 2006). The fact, therefore, that *A. hyacinthus* and *A. digitifera* did not spawn at this time in Pulau Tioman is surprising as these species have been observed to spawn during the major multi-species spawning period in April in nearby Singapore (Guest et al., 2005a) and Bintan, Indonesia (unpublished data). All sampled colonies were >30 cm in diameter and were found in the same habitat, thus ruling out the possibility that sampled colonies were reproductively immature. It is also interesting to note that the lunar timing of spawning is earlier in Pulau Tioman than for conspecifics in Singapore. For example most species in Singapore spawn between 3 and 6 nights after the full moon (Guest et al., 2002; Guest et al., 2005b) whereas in Pulau Tioman corals spawned between 2 nights before and 2 nights after the full moon.

Clearly, more research is needed to determine the timing and extent of coral spawning synchrony on these reefs and elsewhere in Malaysia. In particular, year-round sampling is required to establish reproductive phenologies for a range of species to determine the cause of timing differences within conspecifics among locations. Comparisons of spawning timing among reefs between the east and west coasts of Peninsular Malaysia are of particular interest, as they experience contrasting monsoon seasons and environmental conditions (Toda et al., 2007).

## Additional Information and Declarations

### Field Study Permissions

The following information was supplied relating to field study approvals (i.e., approving body and any reference numbers): All research work was carried out under a memorandum of understanding between Reef Check Malaysia and the Department of Marine Parks Malaysia.
